# Site-Specific Gut Microbiome Changes After Roux-en-Y Gastric Bypass in Rats: Effects of a Multicomponent Bovine Colostrum-Based Complex

**DOI:** 10.3390/ijms26157186

**Published:** 2025-07-25

**Authors:** Zhanagul Khassenbekova, Kadyrzhan Makangali, Aruzhan Shoman, Assem Sagandyk, Nurislam Mukhanbetzhanov, Farkhad Tarikhov, Timur Fazylov, Ylham Annaorazov, Elizaveta Vinogradova, Samat Kozhakhmetov, Almagul Kushugulova

**Affiliations:** 1 Innovative Center ArtScience, Astana Z00T3X6, Kazakhstan; zhanagul.khassenbekova@gmail.com (Z.K.); nurislam.mukhanbetzhanov@nu.edu.kz (N.M.); timson1193@mail.ru (T.F.); ylkham.annaorazov@gmail.com (Y.A.); 2 Department of Technology of Food and Processing Industry, S. Seifullin Kazakh Agrotechnical Research University, Astana Z11F9K5, Kazakhstan; k.makangali@kazatu.kz (K.M.); shoman.aruzhan0403@gmail.com (A.S.); assema.bukeyeva@gmail.com (A.S.); 3 Laboratory of Microbiome, Center for Life Sciences, National Laboratory Astana, Nazarbayev University, 53 Kabanbay Batyr Ave., Block S1, Astana Z05H0P9, Kazakhstan; farkhad.tarikhov@nu.edu.kz (F.T.); st.paulmississippi@gmail.com (E.V.); akushugulova@nu.edu.kz (A.K.); 4 Department of Surgery and Anesthesiology-Intensive Care, Khoja Akhmet Yassawi International Kazakh-Turkish University, Shymkent X83B2G1, Kazakhstan; 5 Interdisciplinary Sports Research, Center for Genetics and Life Sciences, Sirius University of Science and Technology, 1 Olympic Ave., Sirius Federal Territory, Sochi 354340, Russia

**Keywords:** Roux-en-Y gastric bypass, gut microbiome, site specificity, bovine colostrum, *Enterobacteriaceae*, SCFA-producing bacteria, metabolic syndrome, bariatric surgery, dysbiosis

## Abstract

Roux-en-Y gastric bypass (RYGB) surgery induces profound gut microbiota alterations that may impact metabolic outcomes. This study investigated site-specific effects of a multicomponent bovine colostrum-honey-serviceberry (CHJ) complex on post-RYGB microbiome changes in obese rats. Twenty-nine Wistar rats underwent RYGB surgery with CHJ supplementation, followed by mucosal-associated microbiota analysis from five gastrointestinal segments using 16S rRNA sequencing and serum metabolite profiling. RYGB caused regional-specific changes: decreased alpha diversity, systematic *Proteobacteria* increases (31.2 ± 5.1% in duodenum), and reductions in SCFA-producing bacteria (*Romboutsia*, *Roseburia*). CHJ supplementation exhibited dual effects on the microbiome: restoration of beneficial bacteria (*Lactobacillus*, *Bifidobacterium*) in distal segments while concurrently promoting Enterobacteriaceae growth in proximal regions. CHJ also maintained alpha diversity levels of the mucosa-associated microbiota comparable to those observed in the control group. Disconnects emerged between predicted microbial functions and systemic metabolites: thiamine pathway activation accompanied 78.5% serum vitamin B1 reduction, indicating severe absorption deficits. Three distinct patterns emerged: pro-inflammatory (proximal), decolonization (widespread *Helicobacteraceae* loss), and restorative (selective CHJ-mediated recovery). Results demonstrate that post-RYGB dysbiosis exhibits profound regional heterogeneity requiring segment-specific interventions and highlight complex interactions between nutritional supplementation and surgically altered gut ecology in determining metabolic outcomes.

## 1. Introduction

Metabolic syndrome (MetS) is a complex disorder characterized by obesity, insulin resistance, hypertension, and dyslipidemia. The global prevalence of MetS varies from 12.5% to 31.4% depending on the diagnostic criteria used, affecting approximately 25% of the world’s population [1,2]. In recent years, there has been growing interest in the role of the gut microbiome in MetS development and progression [3], with the microbiota influencing metabolic health through the production of bioactive metabolites such as short-chain fatty acids [4,5].

Bariatric surgery, particularly Roux-en-Y gastric bypass (RYGB), has emerged as the most effective treatment for severe obesity and associated MetS. While RYGB leads to significant weight loss and metabolic improvements, recent studies have revealed profound and sometimes concerning changes in gut microbiota composition [6,7]. Recent multi-cohort validation studies have established robust, reproducible microbiome signatures following RYGB that persist across diverse populations, with consistent increases *in Veillonella*, *Streptococcus*, *Gemella*, *Fusobacterium*, *Escherichia*/*Shigella*, and *Akkermansia* across multiple studies [8]. These alterations extend beyond simple taxonomic shifts to include functional changes in microbial metabolism and host–microbe interactions.

Post-RYGB microbial alterations are characterized by decreased alpha diversity, dramatic increases in *Proteobacteria* (particularly *Enterobacteriaceae*), and significant reductions in beneficial butyrate-producing bacteria [7]. Critically, recent research demonstrates that 96.2% of increased *γ-Proteobacteria* after RYGB are *Enterobacteriaceae* members [9]. A recent systematic review by Davies et al. (2019) [10] highlighted that these changes may persist for months after surgery, though some alterations tend to regress to presurgery levels by 12 months. Sorysz et al. (2025) recently emphasized that post-surgical complications, including small intestinal bacterial overgrowth (SIBO), may be linked to these microbial changes [11].

The surgically altered gastrointestinal anatomy creates unique challenges for maintaining healthy gut microbiota. Recent research has shown that the bypassed duodenum and proximal jejunum experience altered pH gradients, reduced exposure to gastric acid, and direct contact with bile acids, creating an environment favoring the growth of facultative anaerobes and potentially pathogenic bacteria [11]. Moreover, the regional heterogeneity of these changes has only recently been appreciated, with different intestinal segments showing distinct microbial signatures post-surgery [12].

Nutritional interventions to modulate post-surgical dysbiosis have gained attention. Bovine colostrum, rich in immunoglobulins, lactoferrin, growth factors, and antimicrobial peptides, has shown promise in supporting gut health. Recent studies demonstrate that colostrum can enhance the growth of beneficial bacteria like *Lactobacillus* and *Bifidobacterium* while potentially inhibiting pathogens. Yang et al.’s 2025 study demonstrated that bovine colostrum supplementation increases gut microbiota diversity while significantly reducing *Enterococcus* abundance and improving intestinal barrier function, with anti-inflammatory properties and prebiotic effects particularly relevant for post-surgical recovery [13]. However, Elsasser et al. (2023) [14] noted that colostrum’s effects on the microbiome are highly context-dependent and may vary based on the host’s physiological state. Understanding the complex interactions between nutritional interventions and host signaling pathways is crucial, as recent research has shown how host-derived factors can modulate gut barrier function and microbial communities in inflammatory conditions [15].

The combination of colostrum with other bioactive compounds may enhance its effects. Honey contains oligosaccharides with prebiotic properties that reduce pathogenic bacteria (*Salmonella*, *E. coli*, *C. difficile*) while stimulating beneficial *Lactobacillus* and *Bifidobacteria* species through non-digestible oligosaccharides [16], while serviceberry (*Amelanchier*) provides polyphenols with antioxidant and anti-inflammatory activities. Recent research suggests that such multicomponent complexes may have synergistic effects on the gut microbiome through various mechanisms, including prebiotic effects, antimicrobial activity, and modulation of host immune responses.

Importantly, recent studies have highlighted potential risks of nutritional interventions in the post-bariatric surgery setting. The altered intestinal anatomy may change how supplements interact with the gut microbiota, potentially leading to unexpected outcomes, including overgrowth of opportunistic pathogens [17]. Chen et al.’s 2024 study identified a novel bile acid-microbiome pathway where sleeve gastrectomy increases cholic acid-7-sulfate production through lithocholic acid activation of the vitamin D receptor, mechanistically connecting microbial metabolites to diabetic phenotype improvements [18]. A 2023 study using fecal microbiota transplantation demonstrated that post-RYGB microbiome changes can directly influence metabolic outcomes, emphasizing the critical role of microbiota in surgical success [19].

The present study aims to investigate the region-specific effects of a multicomponent natural complex based on bovine colostrum with honey and serviceberry (CHJ) on the gut microbiome of rats with metabolic syndrome that have undergone RYGB. We hypothesize that this complex may modulate post-surgical microbial changes differently across intestinal regions, with the potential for both beneficial and adverse effects depending on the local microenvironment. Understanding these regional responses is crucial for developing targeted interventions to optimize post-surgical outcomes while minimizing the risks of dysbiosis-related complications.

## 2. Results

Following obesity model establishment, the average weight of animals was: OB-RYGB—401.0 ± 22.9 g, CHJ-RYGB—403.8 ± 16.8 g, corresponding to target parameters for bariatric intervention. Control groups demonstrated body weights of: CN/SD—219.1 ± 30.1 g, CHJ-control—217.1 ± 7.2 g.

Following tissue collection and microbiome analysis, a total of 26,923,673 reads were processed, with 22,805,575 reads (96,397 end-trimmed) accepted as high quality and 4,118,098 reads rejected during quality filtering. After filtering, 8485 ASVs (Amplicon Sequence Variants), 24,816,874 reads remained in the final matrix, with an average sequencing depth of 24,239,271 reads per sample. The mean depth of the taxonomic tables was 175,760 ± 31,323.

**Figure 1:**
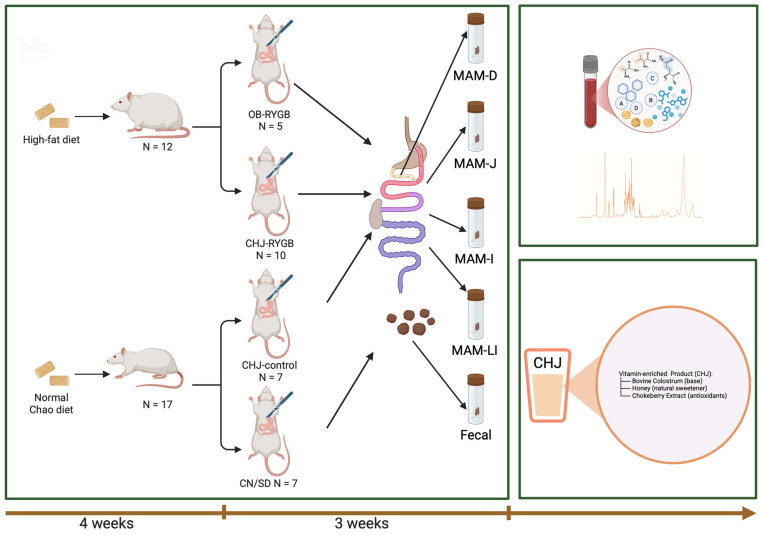
Flowchart of the animal experiment.

**Figure 2:**
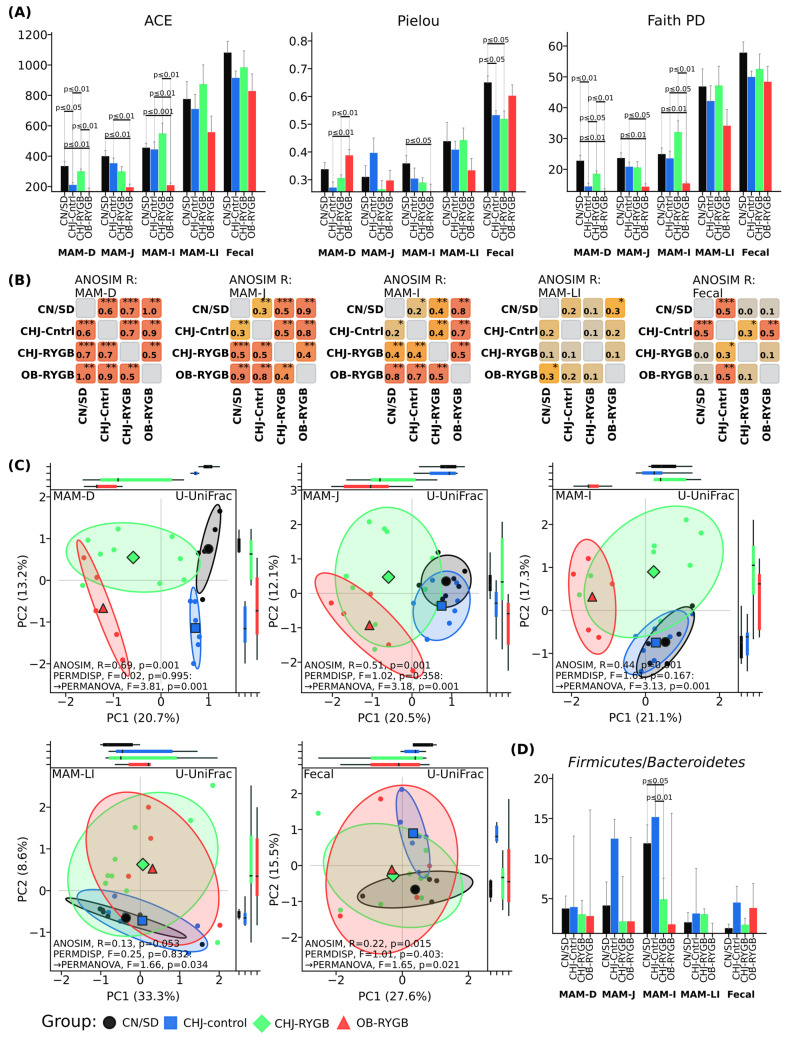
Microbiome diversity and composition changes following RYGB surgery. (**A**) Alpha diversity indices (ACE, FaithPD, Pielou) showing significant reductions after RYGB, with CHJ supplementation restoring diversity levels. (**B**) Pairwise ANOSIM dissimilarity matrices showing intragroup dissimilarities by segment. (**C**) Principal Coordinates Analysis (PCoA) based on unweighted UniFrac distances demonstrating regional specificity of microbiome changes. (**D**) Firmicutes/Bacteroidetes ratio changes across gastrointestinal segments. Groups: CN/SD—lean control; CHJ-control—animals receiving CHJ; CHJ-RYGB—post-RYGB animals receiving CHJ; OB-RYGB—post-RYGB obese animals without supplementation. Statistical significance: * *p* < 0.05, ** *p* < 0.01, *** *p* < 0.001.

RYGB surgery resulted in a significant reduction of microbial diversity, systemic increase in opportunistic pathogenic microflora, and disruption of microbiome metabolic functions. CHJ application partially compensated for these changes, restoring probiotic strains and improving the functional profile of the microbial community (Figure 1 and Figure 2).

The data demonstrate a pronounced reduction in diversity indices in the OB-RYGB group compared to control groups, particularly in the small intestine (MAM-D, MAM-J, MAM-I). The most significant differences are observed for the ACE index in the ileum (MAM-I), where the OB-RYGB group shows substantially lower values (*p* < 0.001) compared to CN/SD. A similar trend is noted for Pielou and Faith PD indices in this intestinal segment.

CHJ application (CHJ-RYGB group) contributes to preservation of diversity levels approximating control values across all studied indices (ACE, Pielou, Faith PD). Statistically significant differences between CHJ-RYGB and OB-RYGB groups indicate a protective effect of CHJ on microbial diversity following bariatric surgery.

In fecal samples, differences between groups in diversity indices are less pronounced, indicating regional specificity of RYGB and CHJ effects on intestinal microbiota.

Beta diversity analysis using principal coordinate analysis (PCoA) based on unweighted UniFrac index showed clear separation of samples by groups with pronounced regional specificity along the GI tract (Figure 2D). ANOSIM grouping assessment demonstrated statistically significant differences in microbial community structure between groups, with the most pronounced changes in proximal segments: duodenum (R = 0.69, *p* = 0.001), jejunum (R = 0.51, *p* = 0.001), and ileum (R = 0.44, *p* = 0.001). Distal segments showed less pronounced differences: large intestine (R = 0.13, *p* = 0.053) and fecal samples (R = 0.22, *p* = 0.015). The CHJ-RYGB group occupied an intermediate position between OB-RYGB and CHJ-control in all studied segments, which can be observed in ANOSIM pairwise comparison matrices (Figure 2B).

### 2.1. Systemic Microbiome Changes Following RYGB

NMR analysis of the multicomponent bovine colostrum-honey-serviceberry (CHJ) complex revealed the following vitamin composition (mg/kg): Vitamin A—6.7679, Vitamin K—19.1025, Vitamin C—28.2856, B1 (thiamine)—1.6397, B2 (riboflavin)—23.0202, B5 (pantothenic acid)—8.0072, B6 (pyridoxine)—0.4563.

**Figure 3:**
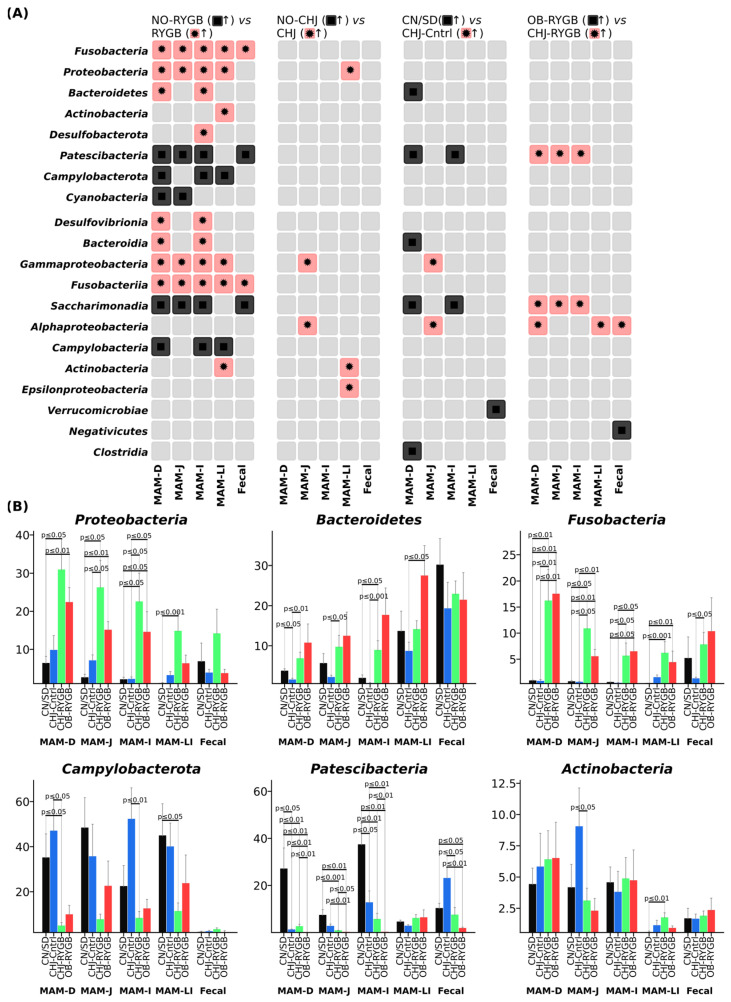
Taxonomic shifts in gut microbiome at phylum level following RYGB surgery. (**A**) Heatmap showing differential abundance patterns across comparisons between groups at phylum and class levels. (**B**) Relative abundance (%) of major bacterial phyla across gastrointestinal segments. RYGB surgery resulted in significant increases in *Proteobacteria*, *Bacteroidetes*, and *Fusobacteria*, while decreasing *Campylobacterota* and *Patescibacteria* in region-specific patterns. CHJ supplementation showed modulatory effects on these taxonomic shifts. Groups: CN/SD—lean control; CHJ-control—obese animals receiving CHJ; CHJ-RYGB—post-RYGB animals receiving CHJ; OB-RYGB—post-RYGB animals without supplementation. ↑: non-overlapping 95% CI.

RYGB surgery resulted in significant taxonomic changes in microbiome composition at the phylum level across all studied GI tract segments (Figure 3B). The most pronounced changes were observed in the phylum *Proteobacteria*, which demonstrated systemic increases following RYGB, particularly in proximal segments, with the most pronounced changes in the *Enterobacteriaceae* family. Statistical analysis showed that this increase was statistically significant across all GI segments (*p* < 0.001–0.05), with the greatest effects in the duodenum. CHJ application enhanced these changes to varying degrees depending on the GI segment.

The phylum *Bacteroidetes* showed increases in distal segments, *Fusobacteria* demonstrated significant growth, while Actinobacteria exhibited moderate changes with regional specificity. Concurrently, decreases in *Campylobacterota* and *Patescibacteria* phyla were observed, indicating profound restructuring of the microbial community. Differential analysis revealed regional specificity of these changes, with different response patterns to CHJ therapy depending on anatomical location.

The *Firmicutes*/*Bacteroidetes* ratio, a classical marker of obesity and metabolic disorders, remained relatively stable across all studied groups (Figure 2D). Only minor variations in this parameter were observed, without pronounced differences between control groups and post-bariatric intervention groups. The CHJ-RYGB group showed intermediate values; however, no statistically significant changes in the overall ratio of these major bacterial phyla were noted.

Differential abundance analysis revealed clear patterns of changes between groups: NO-RYGB vs. RYGB comparisons identified the most pronounced shifts in *Proteobacteria* and *Fusobacteria* phyla, while comparisons with CHJ therapy (OB-RYGB vs. CHJ-RYGB) demonstrated partial normalization of microbial profiles in certain taxonomic groups (Figure 3A).

### 2.2. Changes in Metabolically Active Bacteria

Statistical analysis confirmed significant changes in key functional bacterial groups following RYGB surgery and CHJ application. The analysis revealed three distinct patterns of microbial response along the gastrointestinal tract.

Short-chain fatty acid (SCFA)-producing bacteria showed systemic reduction following RYGB surgery, which was partially compensated by CHJ therapy. *Romboutsia*, a key acetate producer, demonstrated significant reduction after RYGB across all segments, with the most pronounced decrease in the duodenum (CN/SD vs. OB-RYGB: −2.0%, *p* ≤ 0.05) and large intestine (−0.2%, *p* ≤ 0.01). CHJ application provided significant recovery, particularly in the ileum (CHJ-RYGB vs. OB-RYGB: +0.5%, *p* ≤ 0.01). *Roseburia* spp., important butyrate producers, showed similar patterns with postoperative reduction (CN/SD vs. OB-RYGB in ileum: median difference −0.1%, *p* ≤ 0.01) and partial CHJ-mediated recovery (CHJ-RYGB vs. OB-RYGB: +0.1%, *p* ≤ 0.01).

**Figure 4:**
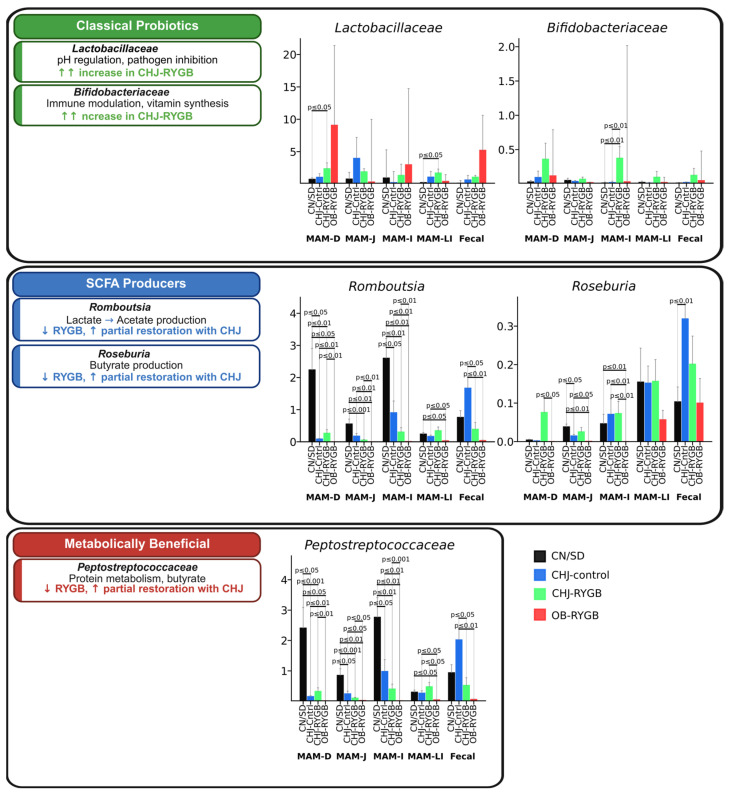
Effects of CHJ supplementation on functionally important bacterial groups following RYGB surgery. Changes in classical probiotics (green), SCFA producers (blue), and metabolically beneficial bacteria (red) across five gastrointestinal segments. Data shown as relative abundance (%) with statistical significance indicators.

The *Peptostreptococcaceae* family, representing metabolically beneficial bacteria involved in protein metabolism and secondary bile acid production, exhibited complex regional variations. Significant reductions were observed in the ileum (CN/SD vs. OB-RYGB: −2.7%, *p* ≤ 0.01), while CHJ therapy showed restoration of *Peptostreptococcaceae* levels with varying effect magnitude depending on intestinal segment, indicating region-specific therapeutic responses (Figure 4).

### 2.3. Effects of Multicomponent Natural Complex on Probiotic Bacteria

Classical probiotic bacteria demonstrated a significant positive response to CHJ application. *Lactobacillus* showed pronounced increases in CHJ-receiving groups, with the most notable effects in the duodenum (CN/SD vs. CHJ-RYGB: +0.3%, *p* ≤ 0.05) and ileum (+1.3%, *p* ≤ 0.01). Similarly, bifidobacteria demonstrated consistent increases across multiple segments, particularly in the large intestine, where CHJ application resulted in a +0.2% increase compared to control (*p* ≤ 0.01) (Figure 4).

### 2.4. Site Specificity of Microbial Changes

**Figure 5:**
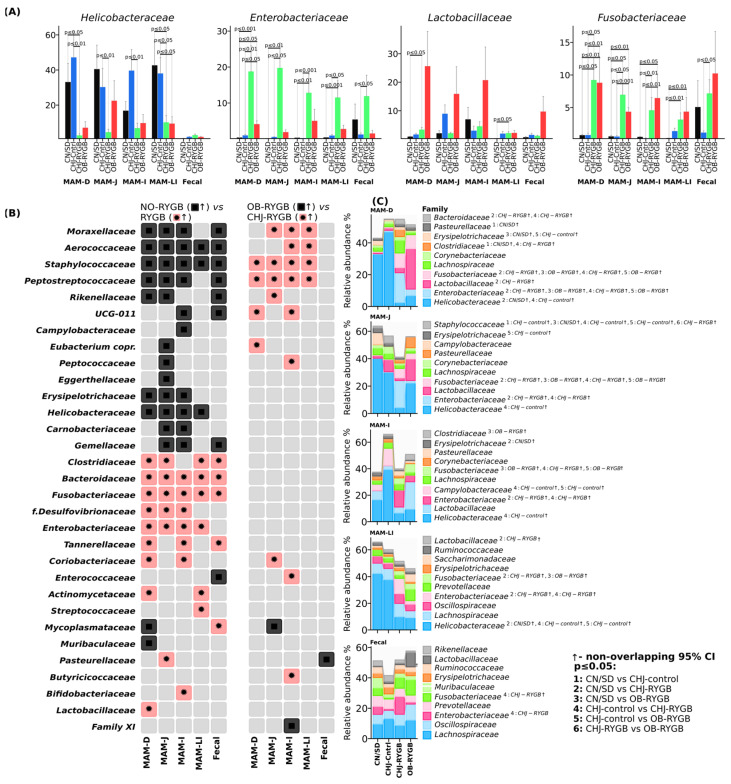
Site-specific changes in gut microbiome composition at the family level across different gastrointestinal segments following RYGB surgery and CHJ supplementation. (**A**) Relative abundance (%) of the most abundant bacterial families. (**B**) Heatmap showing differential abundance patterns across comparisons between groups at family level. (**C**) Relative abundance (%) of major bacterial families in duodenum (MAM-D), jejunum (MAM-J), ileum (MAM-I), large intestine (MAM-LI), and fecal samples. Groups: CN/SD—control without obesity (*n* = 7); CHJ-control—obese animals receiving CHJ (*n* = 7); CHJ-RYGB—post-RYGB animals receiving CHJ (*n* = 10); OB-RYGB—post-RYGB animals without supplementation (*n* = 5). Arrows indicate statistically significant differences with non-overlapping 95% CI, *p* < 0.05.

The duodenum showed the greatest sensitivity to RYGB, with maximal *Proteobacteria* increases from 6.3% to 30.8% in CHJ-RYGB and dramatic Enterobacteriaceae growth (from 0.3% to 18.7% in CHJ-RYGB) (Figure 5). Statistical analysis confirmed high significance of these changes: CN/SD vs. CHJ-RYGB showed a 27.8% increase (*p* ≤ 0.001), while CN/SD vs. OB-RYGB showed a 3.7% increase (*p* ≤ 0.05) (Figure 5C).

The jejunum occupied an intermediate position with moderate *Enterobacteriaceae* increases to 19.7% in CHJ-RYGB (CN/SD vs. CHJ-RYGB +15.9%, *p* ≤ 0.01) while maintaining a significant proportion of *Lachnospiraceae* (2.5%) and pronounced reduction of *Helicobacteraceae* from 40.3% to 4.4% (CN/SD vs. CHJ-RYGB: −24.8%, *p* ≤ 0.01).

The ileum was characterized by significant *Enterobacteriaceae* increases (CN/SD vs. CHJ-RYGB: +12.0%, *p* ≤ 0.01) (Figure 5) and the greatest *Bacteroidetes* changes (CN/SD vs. CHJ-RYGB: +7.0%, *p* ≤ 0.05).

The large intestine showed substantial compositional shifts in the microbiome with decreased representation of SCFA-producing families (*Lachnospiraceae*, *Ruminococcaceae*) and significant *Enterobacteriaceae* increases (CN/SD vs. CHJ-RYGB: +9.5%, *p* ≤ 0.001), indicating potential reduction in short-chain fatty acid production (Figure 5A,C).

Fecal samples confirmed the systemic nature of changes with *Fusobacteriaceae* dominance (NO-RYGB vs. RYGB: +4.5%, *p* ≤ 0.01), reflecting profound disruptions of the intestinal ecosystem (Figure 5).

The analysis revealed three main patterns: pro-inflammatory (proximal segments with Enterobacteriaceae dominance), decolonization (sharp reduction of *Helicobacteraceae* from 32.9–40.3% to 2.5–6.6% across all segments), and restorative (CHJ selectively restored beneficial populations in distal segments while simultaneously increasing opportunistic pathogenic forms in proximal segments).

### 2.5. Predicted Metabolic Functions of the Microbiome and Correlations with Systemic Metabolism

**Figure 6:**
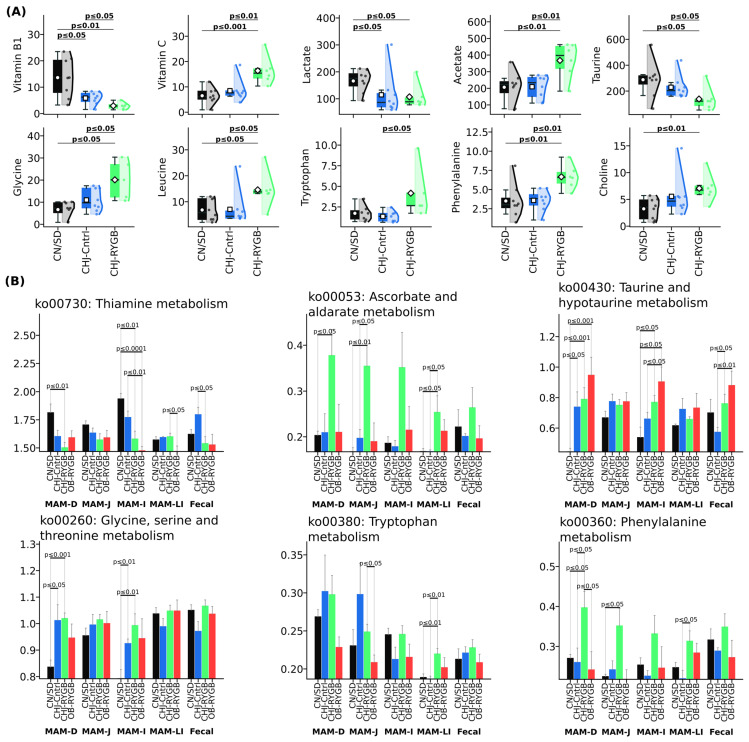
Predicted metabolic functions of gut microbiome and correlations with systemic metabolism. (**A**) Serum levels and metabolite concentrations in groups. (**B**) PICRUSt2 analysis of predicted metabolic pathways showing changes in vitamin B metabolism (ko00730—Thiamine metabolism), amino acid metabolism (ko00260, ko00360, ko00380), and folate biosynthesis (ko00790) across gastrointestinal segments.

Analysis of predicted metabolic capabilities (PICRUSt2) revealed significant changes in the functional profile of the microbiome following RYGB, which were accompanied by pronounced changes in blood serum metabolites (Figure 6). The most pronounced changes were observed in B vitamin metabolism pathways, particularly ko00730 (Thiamine metabolism), which showed reduction in the ileum (CN/SD vs. CHJ-RYGB: *p* ≤ 0.01; CN/SD vs. OB-RYGB: *p* ≤ 0.0001) and duodenum (CN/SD vs. CHJ-RYGB: *p* ≤ 0.01). The reduction in functional potential for microbial B1 synthesis was accompanied by a significant decrease in vitamin B1 concentration in blood serum, which demonstrated pronounced reduction by 78.5% (CN/SD: 13.62 ± 6.95 vs. CHJ-RYGB: 2.93 ± 1.19 μM, *p* < 0.01), also indicating critical impairment of vitamin absorption following RYGB.

RYGB induced characteristic changes in microbial amino acid metabolism. Increased metabolic potential for ko00260 (Glycine, serine, and threonine metabolism) in the duodenum (CN/SD vs. CHJ-RYGB: *p* ≤ 0.001; CN/SD vs. CHJ-control: *p* ≤ 0.05) and ileum (CN/SD vs. CHJ-RYGB: *p* ≤ 0.01; CN/SD vs. CHJ-control: *p* ≤ 0.01) was accompanied by elevated glycine concentration in blood serum (CN/SD vs. CHJ-RYGB: *p* ≤ 0.05; CN/SD vs. CHJ-control: *p* = 0.07). Simultaneously, a statistically significant reduction in taurine by 51.9% was observed (CN/SD vs. CHJ-RYGB: *p* ≤ 0.05; CN/SD vs. CHJ-control: *p* = 0.19).

Activation of tryptophan metabolism was manifested by increased ko00380 (Tryptophan metabolism) in the large intestine (CN/SD vs. CHJ-RYGB: *p* ≤ 0.01; CHJ-control vs. CHJ-RYGB: *p* ≤ 0.01), which was accompanied by elevated tryptophan levels in blood serum (CHJ-control: 1.73 ± 0.87 vs. CHJ-RYGB: 4.17 ± 2.83 μM, *p* ≤ 0.05), potentially indicating activation of the serotonin pathway following RYGB.

Despite reduced abundance of key SCFA-producing bacteria in the microbiome, serum acetate concentration increased by 78.1% (CN/SD: 206.36 ± 88.22 vs. CHJ-RYGB: 367.5 ± 99.32 μM, *p* < 0.01), which may reflect compensatory mechanisms. Lactate concentration also showed an increase (*p* < 0.05).

The increased vitamin C concentration in the CHJ-RYGB group (CN/SD: 6.5 ± 3.03 vs. CHJ-RYGB: 16.38 ± 5.12 μM, *p* < 0.001) reflects the direct ascorbic acid content in the CHJ multicomponent complex. Activation of pathway ko00053 (Ascorbate and aldarate metabolism) in the large intestine (CN/SD vs. CHJ-RYGB: *p* = 0.018511; CHJ-control vs. CHJ-RYGB: *p* = 0.022478) may reflect adaptive microbiome changes. It is important to note that vitamin C is not synthesized by mammalian intestinal microbiota; therefore, this increase cannot be related to activation of microbial synthesis.

It is worth noting that although significant systematic changes were observed in the functional and metabolite profiles between the control and RYGB groups, no direct significant correlations (Spearman’s, *p* > 0.05) were observed between metabolite levels and functional profiles. This disconnect suggests that the relationship between predicted microbial functions and systemic metabolites is complex and may involve additional factors beyond simple linear correlations, including altered absorption kinetics, host–microbe metabolic interactions, and the surgically modified gastrointestinal anatomy.

## 3. Discussion

The present study demonstrated profound changes in the structure and function of the rat intestinal microbiome following RYGB surgery, which is consistent with current understanding of the impact of bariatric surgery on intestinal microbial ecology. Our observed reduction in alpha diversity in the OB-RYGB group by ACE and Faith PD indices in the ileum (*p* < 0.001 and *p* < 0.01, respectively) corresponds to data from other studies showing similar changes after gastrointestinal operations [20]. A critically important result is the identified regional specificity of changes through beta diversity analysis using ANOSIM test, which showed the most pronounced changes in proximal segments: duodenum (R = 0.69, *p* = 0.001), jejunum (R = 0.51, *p* = 0.001) and ileum (R = 0.44, *p* = 0.001), while distal segments showed less pronounced differences: large intestine (R = 0.13, *p* = 0.053) and fecal samples (R = 0.22, *p* = 0.015).

Our results fully align with recent multi-cohort validation studies that established robust, reproducible microbiome signatures following RYGB, persisting across diverse populations and successfully discriminating pre- and post-surgical samples based on pathogenic signatures [8]. Surgical reconstruction in RYGB creates unique segment-specific environments where bile acids serve as key signaling molecules, activating intestinal FXR and systemic TGR5 pathways [21]. Elevated concentrations of conjugated bile acids in the biliopancreatic limb explain the preferential growth of bile-resistant Enterobacteriaceae in proximal segments, corresponding to our observations of maximal increases up to 31.2 ± 5.1% in the duodenum. Reduction in parietal cell numbers in the small gastric pouch decreases acidity from pH 1.5 to 3–4, creating favorable conditions for facultative anaerobes and acid-sensitive bacteria [22]. This mechanism explains the systemic increase in Proteobacteria, especially in proximal segments, where the effect is most pronounced.

Particularly noteworthy is the systemic increase in *Proteobacteria* phylum representatives, whose abundance increased 3–4-fold after RYGB. This is consistent with the study by Prykhodko et al. (2024), who also observed significant *Proteobacteria* increases after RYGB in humans [6]. The mechanism of this phenomenon may be related to pH changes in proximal intestinal segments due to reduced gastric acid secretion and altered food passage pathways [7].

The most concerning result of our study is the increase in *Enterobacteriaceae* family, particularly in the duodenum (from 0.3% to 18.7% in the CHJ-RYGB group, *p* < 0.001). Significant increases were observed across all segments: jejunum (CN/SD vs. CHJ-RYGB, *p* < 0.01), ileum (CHJ-control vs. CHJ-RYGB, *p* < 0.05), large intestine (CHJ-control vs. CHJ-RYGB, *p* < 0.001). This increase represents a serious risk for developing infectious complications. Similar results were described by Sorysz et al. (2025), who also noted significant *Enterobacteriaceae* increases after RYGB in patients [11]. SIBO affects 40–43% of patients after RYGB compared to 15% before surgery, which is attributed to the creation of blind loops and impaired motility [23]. Our study demonstrates analogous patterns with dramatic increases in Enterobacteriaceae in proximal segments, which may serve as a predictor for SIBO development. Interestingly, in our study, CHJ application enhanced this effect, leading to even greater *Proteobacteria* increases (up to 31.2 ± 5.1% in the duodenum). This may be related to the fact that colostrum components, while possessing probiotic properties, under altered GI tract anatomy conditions after RYGB may create favorable conditions for facultative anaerobe growth, including Enterobacteriaceae [24].

The increase in *Proteobacteria* and *Enterobacteriaceae* may be related to increased oxygen availability in proximal intestinal segments after RYGB, creating favorable conditions for facultative anaerobes. This is confirmed by recent studies showing that changes in intestinal microaeration after bariatric surgery promote the growth of aerotolerant bacteria [25].

One of the key results of our study is the systemic reduction in SCFA-producing bacteria abundance. *Romboutsia ilealis* showed statistically significant reduction across all GI tract segments, with the most pronounced decrease in the duodenum (CN/SD vs. OB-RYGB, *p* < 0.05) and large intestine (*p* < 0.01). CHJ application provided significant recovery, particularly in the large intestine (CHJ-RYGB vs. OB-RYGB, *p* = 0.01). *Roseburia* spp. showed similar patterns with dramatic postoperative reduction (CN/SD vs. OB-RYGB in large intestine, *p* < 0.01) and partial CHJ-mediated recovery (CHJ-RYGB vs. OB-RYGB, *p* < 0.01). These data correspond to results from Hamamah et al. (2024), who also noted a reduction in butyrate-producing bacteria after RYGB [7], Supplementary Figure S1.

Despite reduced SCFA producer abundance, blood serum acetate concentration increased by 78.1% in our study. This may be explained by compensatory mechanisms or changes in SCFA absorption under altered intestinal anatomy conditions. Similar results were obtained in the study by Juárez-Fernández et al. (2021), where increased concentrations of certain SCFAs in blood were also observed despite reduced fecal concentrations [17].

Our study is the first to characterize in detail the regional specificity of microbiome changes along the entire GI tract length after RYGB. The duodenum showed the greatest sensitivity to surgical intervention, with maximal Proteobacteria increases and dramatic Enterobacteriaceae growth. This may be related to direct contact of this segment with bile and pancreatic enzymes without prior neutralization by gastric juice [26].

The analysis revealed three main patterns of microbial changes: pro-inflammatory (proximal segments with *Enterobacteriaceae* dominance), decolonization (sharp *Helicobacteraceae* reduction from 32.9–40.3% to 2.5–6.6% across all segments), and restorative (CHJ selectively restored beneficial populations in distal segments while simultaneously increasing opportunistic pathogenic forms in proximal segments). Contemporary machine learning approaches enable prediction of RYGB success based on microbiome profiles, opening possibilities for personalized therapeutic approaches [8]. Our results regarding three distinct patterns of changes (pro-inflammatory, decolonization, and restorative) may serve as a foundation for such predictive models. Development of spatial microbiomics technologies allows mapping of changes at specific anatomical locations, confirming the importance of our segment-specific approach and the necessity for targeted interventions [27].

In the large intestine, the most pronounced changes in SCFA producers were observed, which may have long-term consequences for metabolic health. These data complement results from Palmisano et al. (2020), who also showed regional heterogeneity of microbial changes after bariatric surgery [12].

The CHJ application demonstrated a dual effect on the microbiome. On one hand, partial restoration of alpha diversity was observed, particularly in distal GI tract segments (MAM-LI *p* < 0.05 vs. OB-RYGB). On the other hand, CHJ enhanced the growth of opportunistic pathogenic microorganisms in the proximal segments.

A positive effect of CHJ was a significant increase in classical probiotic strain abundance. *Lactobacillaceae* showed systemic increases in the CHJ-RYGB group, with the most notable effects in the duodenum (*p* < 0.05) and ileum (*p* < 0.01). *Bifidobacteriaceae* demonstrated consistent increases across multiple segments, particularly in the large intestine, where CHJ application resulted in a +5.7% increase compared to control (*p* < 0.05). This is consistent with known probiotic properties of colostrum [13]. A 2025 meta-analysis demonstrated that probiotics significantly reduce BMI (mean difference −0.67 kg/m^2^) when administered perioperatively with optimal doses of 10^9^–10^10^ CFU/day for 3–6 months [28]. This confirms the therapeutic potential of our CHJ complex, particularly in the context of restoring probiotic strains in distal segments. These results are confirmed by recent clinical trials demonstrating promising results for probiotic interventions in post-bariatric patients, with a 2024 umbrella meta-analysis analyzing 11 randomized controlled trials with 559 patients finding significant reductions in waist circumference and body weight, with improved liver function markers (AST reduction: MD = −4.32 U/L). *Lactobacillus* and *Bifidobacterium* strains proved most effective, with an optimal intervention duration of 12 weeks post-surgery [29]. Similar results were obtained for *Bifidobacterium*, confirming data from Elsasser et al. (2023) on colostrum’s ability to support bifidobacteria growth [14].

The observed microbiome changes had pronounced metabolic consequences. Reduction in ko00730 (Thiamine metabolism) in the ileum (CN/SD vs. CHJ-RYGB: *p* = 0.00393; CN/SD vs. OB-RYGB: *p* = 0.000107) and jejunum (CHJ-control vs. OB-RYGB: *p* = 0.004715) was accompanied by critical vitamin B1 reduction in serum by 78.5% (*p* < 0.01), indicating a dual mechanism of thiamine deficiency in RYGB: reduced microbial synthesis combined with absorption impairments. The observed 78.5% reduction in serum vitamin B1 levels despite activation of microbial thiamine synthesis pathways indicates critical absorption impairments independent of microbial production [30]. This is consistent with data showing that anatomical changes create independent B vitamin absorption impairments, emphasizing the need for a multimodal approach to deficit correction. Recent studies show that severe obesity is associated with the absence of biotin-producing bacteria in the gut, creating a vicious cycle where intestinal inflammation limits biotin uptake from food. Bariatric surgery stimulates the growth of biotin-producing bacteria, particularly during the first year after surgery, but anatomical changes create independent absorption impairments [31]. Similar results were described by Davies et al. (2019), who also noted B vitamin deficiencies after RYGB despite changes in microbial metabolism [10].

Increased vitamin C concentration in the CHJ-RYGB group (CN/SD: 6.5 ± 3.03 vs. CHJ-RYGB: 16.38 ± 5.12 μM, *p* < 0.001) was accompanied by activation of pathway ko00053 (Ascorbate and aldarate metabolism) in the large intestine (CN/SD vs. CHJ-RYGB: *p* = 0.018511; CHJ-control vs. CHJ-RYGB: *p* = 0.022478). This may reflect exogenous ascorbic acid intake as part of the CHJ complex rather than activation of microbial synthesis, since similar increases were not observed in the CHJ-control group without surgical intervention. Adaptive changes in microbial metabolism may be related to both direct vitamin C content in the supplement and absorption changes after RYGB.

Notably, in our study, *Akkermansia muciniphila* was not found in significant quantities (less than 0.1% across all groups), contrasting with data from many other studies. For example, Ioannou et al. (2025) and other authors reported significant *A. muciniphila* increases after RYGB, linking this to improved metabolic parameters [32]. The absence of this bacterium in our study may be related to experimental animal diet characteristics or technical aspects of sequencing.

Long-term effects of bariatric surgery on the microbiome remain subject to debate. The study by Shen et al. (2019) showed that changes in alpha diversity and microbiome composition may regress to preoperative levels 12 months after surgery [33]. Microbial changes after RYGB occur rapidly—within 1–3 months—and persist long-term, indicating fundamental restructuring of intestinal ecology [34]. Our study, conducted 21 days post-surgery, captures the early phase of these changes, requiring continuation of long-term observations. However, our study, conducted 21 days after surgery, demonstrates persistent changes that may be early predictors of long-term effects.

Bariatric surgery significantly alters the intestinal inflammatory profile through microbiota modulation. Reduced lipopolysaccharide (LPS) and pro-inflammatory cytokine (IL-1, IL-6, TNF-α) concentrations after RYGB are associated with improved microbiota composition [7].

The obtained results have critical clinical significance for understanding the mechanisms of microbial changes after bariatric surgery. Prophylactic pre- and probiotic interventions beginning 6 weeks before surgery and continuing 3–6 months post-surgery may lead to reduced complications and improved outcomes. Increased opportunistic pathogenic microflora, particularly Enterobacteriaceae, may explain the elevated risk of infectious complications after RYGB described in clinical studies [35].

Application of the colostrum-based multicomponent complex showed potential for partial correction of dysbiotic changes but requires further optimization to prevent enhanced growth of opportunistic pathogenic microorganisms. Synbiotic formulations combining probiotics with prebiotics demonstrate superior metabolic outcomes compared to monotherapy [36]. This supports our conclusion regarding the need for CHJ complex optimization to prevent opportunistic pathogen growth while maintaining probiotic effects. Possibly, combining colostrum with selective prebiotics or postbiotics could provide more balanced microbiome restoration.

Several limitations of our study should be noted. First, using a rat model may not fully reflect changes occurring in humans. Second, the relatively short observation period (21 days post-surgery) does not allow assessment of long-term effects. Third, species-level taxonomic assignments based on V3–V4 16S rRNA amplicon sequencing may have limitations due to the inherent resolution constraints of short-read sequencing platforms. While the V3–V4 region provides adequate resolution for most gut microbiome analyses, some species-level identifications should be interpreted with caution, particularly for closely related taxa. Future studies employing full-length 16S rRNA sequencing using third-generation technologies would provide enhanced taxonomic resolution. Nevertheless, we obtained reliable results showing site-specific microbiome changes after RYGB and the influence of colostrum-based multicomponent complex on beneficial microflora restoration in distal GI tract segments while simultaneously enhancing opportunistic pathogenic microflora growth in proximal segments.

## 4. Materials and Methods

### 4.1. Animal Study

The study utilized 42 Wistar rats. Animals were fed Ssniff^®^ V1534-300 (Ssniff^®^ Spezialdiäten GmbH, Soest, Germany), a standard maintenance diet for laboratory rodents composed of 24.0 kJ% protein, 67 kJ% carbohydrate, and 9 kJ% fat. To establish an obesity model, experimental groups were maintained for 4 weeks on the standard diet supplemented with animal fat (30 g/kg) and fructose in drinking water (10% solution), while control animals received the standard diet with regular water. Rats were housed in plastic cages under a 12 h light-dark cycle at an ambient temperature of 23 °C ± 2 °C. Four groups were formed: OB-RYGB (*n* = 5)—obese animals after RYGB receiving standard feed and water; CHJ-control (*n* = 7)—obese animals receiving standard feed and bovine colostrum containing honey and serviceberry; CHJ-RYGB (*n* = 10)—obese animals after RYGB receiving standard feed and bovine colostrum containing honey and serviceberry; CN/SD (*n* = 7)—control group without obesity. Thirteen animals died in the postoperative period and were excluded from analysis. Final analysis was conducted on 29 animals. This study was conducted and reported in accordance with the ARRIVE (Animal Research: Reporting of In Vivo Experiments) Guidelines 2.0 for reporting animal research.

### 4.2. Preoperative Preparation

Preoperative preparation included overnight fasting and hair removal from the abdominal wall using an electric trimmer. The surgical field was sequentially treated with iodine solution and covered with sterile surgical drapes. Anesthesia was performed using sevoflurane inhalation.

### 4.3. Surgical Procedure and Postoperative Care

The Roux-en-Y gastric bypass (RYGB) procedure was performed according to established protocol [37]. Postoperatively, animals were housed in individual cages. During the first three days after surgery, rats received a liquid diet with a gradual transition to a standard diet, which was fully restored by day 7. Food consumption volume was standardized across all experimental groups. Twenty-one days after surgery, mucosal-associated microbiota samples were collected from four GI tract segments: jejunum (MAM-J), ileum (MAM-I), large intestine (MAM-LI), duodenum (MAM-D), and fecal samples.

### 4.4. Sample Processing and DNA Extraction

Mucosal-associated microbiota samples were collected from each animal from the duodenum, jejunum, ileum, large intestine, and feces. During surgical procedures, each sample was placed in a microtube and immediately frozen at −70 °C. Sample transport was performed at −20 °C.

DNA extraction was performed using the ZymoBiomics DNA Microprep kit (Cat. No.: D4300, Zymo Research Corporation, Irvine, CA, USA). For standardized DNA extraction, exactly 0.2 mL of sample was used. Quality of extracted total DNA was verified by electrophoresis in 1% agarose gel, and DNA concentration was measured using a Nabi UV/Vis Nano spectrophotometer (MicroDigital, Gyeonggi-do, Seongnam, Republic of Korea).

Then, 16S rRNA gene amplicon sequencing targeting the V3–V4 hypervariable regions was performed using standard Illumina library preparation protocols on the NovaSeq6000 platform by Novogene (Beijing, China). Universal primers 341F: CCTAYGGGRBGCASCAG and 806R: GGACTACNNGGGTATCTAAT were used for V3–V4 region amplification with paired-end sequencing (2 × 250 bp). Sequencing was conducted both internally using the Illumina NovaSeq6000 platform following 16S amplicon sequencing protocols, and externally at Novogene laboratory (Beijing, China), also utilizing the Illumina NovaSeq 6000 platform according to the manufacturer’s standard procedures. Initial bioinformatic analysis was completed using LotuS2 (less operational taxonomic unit scripts 2) [38].

### 4.5. Serum Biochemical Analysis and NMR Methodology

Blood serum samples were analyzed using Nuclear Magnetic Resonance (NMR) spectroscopy to determine the following parameters: amino acid profile including alanine, lysine, valine, leucine, tyrosine, glycine, histidine, glutamate, aspartic acid, asparagine, tryptophan, and phenylalanine; organic acids and metabolites including lactate, acetate, 3-hydroxybutyrate (3-HB), formate, fumarate, choline, allantoin, and urea; vitamins including retinol, ascorbic acid, cholecalciferol, thiamine, riboflavin, pyridoxine, folic acid, and cobalamin; carbohydrates including glucose and N-acetylglucosamine; and lipoproteins including low-density lipoprotein (LDL) and high-density lipoprotein (HDL).

For serum NMR analysis, sample preparation was performed as follows: 300 μL of serum sample was collected and mixed with 300 μL of deuterated water (D2O). The mixture was centrifuged at 12,000× *g* for 10 min at room temperature. Then, 550 μL of supernatant was transferred to a standard 5 mm diameter NMR tube. For chemical shift calibration, 50 μL of 5 mM sodium trimethylsilylpropionate (TSP-d4) solution was added to the sample as an internal standard. All biochemical parameters were quantitatively determined using NMR spectroscopy through integrated peak analysis and chemical shift identification.

Vitamin composition of multicomponent bovine colostrum-honey-serviceberry (CHJ) complex was also analyzed using NMR methodology. Sample preparation of CHJ for NMR analysis was performed as follows: 100 mg of dry CHJ was dissolved in 1 mL of deuterated water (D2O). The mixture was intensively vortexed for 5 min until a homogeneous solution was obtained, then centrifuged at 12,000× *g* for 10 min at room temperature. 550 μL of supernatant was transferred to a standard 5 mm diameter NMR tube.

### 4.6. Microbiome Data Analysis and Statistical Processing

Statistical analyses and visualizations were performed in Python v3.12 using NumPy v2.0.1 [39], SciPy v1.15.1 [40], scikit-bio v0.6.3, Matplotlib v3.10.0 [41], and seaborn v0.13.2 packages [42]. The mean depth of the taxonomic tables was 175,760 ± 31,323, with no significant differences observed across gut sites (*p* = 0.11) or experimental groups (*p* = 0.93). Predicted functional profiles of microbial communities were generated using PICRUSt2 (Phylogenetic Investigation of Communities by Reconstruction of Unobserved States 2, version 2.5.2) [43]. The analysis was performed using the default workflow with ASV sequences placed into a reference phylogeny using EPA-ng, followed by hidden-state prediction to predict gene family abundances and pathway abundance inference using MinPath. KEGG Orthology (KO) functional predictions were generated, and pathway abundances were calculated, normalized by 16S rRNA gene copy number. Differential analysis was performed using relative abundance data. Only taxa prevalent in 100% of the samples in any group-subgroup combination and mean relative abundance higher than 0.01% were considered for the analysis. Differences in abundance were considered significant at *p* ≤ 0.05 and with non-overlapping 95% confidence intervals (CIs) for the differences between central statistics (means or medians, where appropriate), no additional correction for multiple comparisons was performed. Tests for differential analysis were selected based on the distribution of the data. For normally distributed data, a T-test with or without the Welch correction and the CI for the difference in means was used. For non-normally distributed data, the Mann–Whitney U-test with the Hodges–Lehmann estimator for the difference between medians was used. Beta diversity analysis was performed using unweighted and weighted UniFrac distances (U-UniFrac and W-UniFrac, respectively). ANOSIM and PERMANOVA tests with 999 permutations were used to assess the significance of grouping. Before performing the PERMANOVA test, homogeneity of multivariate variances was checked using PERMDISP; variances were considered comparable at *p* > 0.05. Alpha diversity analysis was performed using ACE, Pielou, and Faith indexes. Spearman’s r coefficient was used to perform correlation analysis between marker taxa, pathways, and serum data. For visualization purposes, data were right-hand winsorized, and means with standard error were shown on the barplots.

## 5. Conclusions

Our study demonstrated that RYGB induces profound and site-specific changes in the intestinal microbiome, characterized by increased opportunistic pathogenic microflora, reduced SCFA producers, and functional disruptions of the microbial community. Critically important is the discovery of different dysbiosis patterns across distinct GI tract segments, necessitating personalized therapeutic approaches. Application of the colostrum-based multicomponent complex showed a dual effect: restoration of beneficial microflora in distal segments while simultaneously enhancing opportunistic pathogen growth in proximal segments. These results justify the need for developing segment-specific interventions and underscore the potential of personalized medicine based on individual microbiome profiles.

## Data Availability

Sequence data of the current study have been deposited in the NCBI BioProject; the primary accession number is PRJNA1291440.

## Supplementary Materials

The following supporting information can be downloaded at: https://www.mdpi.com/article/10.3390/ijms26157186/s1.

## References

[1] Noubiap J.J., Nansseu J.R., Lontchi-Yimagou E., Nkeck J.R., Nyaga U.F., Ngouo A.T., Tounouga D.N., Tianyi F.-L., Foka A.J., Ndoadoumgue A.L. (2022). Geographic distribution of metabolic syndrome and its components in the general adult population: A meta-analysis of global data from 28 million individuals. Diabetes Res. Clin. Pract..

[2] Saklayen M.G. (2018). The Global Epidemic of the Metabolic Syndrome. Curr. Hypertens. Rep..

[3] Fan Y., Pedersen O. (2021). Gut microbiota in human metabolic health and disease. Nat. Rev. Microbiol..

[4] Canfora E.E., Jocken J.W., Blaak E.E. (2015). Short-chain fatty acids in control of body weight and insulin sensitivity. Nat. Rev. Endocrinol..

[5] Koh A., De Vadder F., Kovatcheva-Datchary P., Bäckhed F. (2016). From Dietary Fiber to Host Physiology: Short-Chain Fatty Acids as Key Bacterial Metabolites. Cell.

[6] Prykhodko O., Burleigh S., Campanello M., Iresjö B.M., Zilling T., Ljungh Å., Smedh U., Hållenius F.F. (2024). Long-Term Changes to the Microbiome, Blood Lipid Profiles and IL-6 in Female and Male Swedish Patients in Response to Bariatric Roux-en-Y Gastric Bypass. Nutrients.

[7] Hamamah S., Hajnal A., Covasa M. (2024). Influence of Bariatric Surgery on Gut Microbiota Composition and Its Implication on Brain and Peripheral Targets. Nutrients.

[8] Fouladi F., Carroll I.M., Sharpton T.J., Bulik-Sullivan E., Heinberg L., Steffen K.J., Fodor A.A. (2021). A microbial signature following bariatric surgery is robustly consistent across multiple cohorts. Gut Microbes.

[9] Zhang H., DiBaise J.K., Zuccolo A., Kudrna D., Braidotti M., Yu Y., Parameswaran P., Crowell M.D., Wing R., Rittmann B.E. (2009). Human gut microbiota in obesity and after gastric bypass. Proc. Natl. Acad. Sci. USA.

[10] Davies N.K., O’Sullivan J.M., Plank L.D., Murphy R. (2019). Altered gut microbiome after bariatric surgery and its association with metabolic benefits: A systematic review. Surg. Obes. Relat. Dis..

[11] Sorysz Z., Kowalewski P., Walędziak M., Różańska-Walędziak A. (2025). Do Gut Microbiomes Shift After Bariatric Surgery? A Literature Review. Medicina.

[12] Palmisano S., Campisciano G., Silvestri M., Guerra M., Giuricin M., Casagranda B., Comar M., de Manzini N. (2020). Changes in Gut Microbiota Composition after Bariatric Surgery: A New Balance to Decode. J. Gastrointest. Surg..

[13] Yang L., Hui Y., Thymann T., Nielsen D.S., Jiang P.P., Sangild P.T. (2025). Bovine colostrum prevents formula-induced gut microbiota dysbiosis in preterm pigs. Pediatr. Res..

[14] Elsasser T.H., Ma B., Ravel J., Kahl S., Gajer P., Cross A. (2023). Short-term feeding of defatted bovine colostrum mitigates inflammation in the gut via changes in metabolites and microbiota in a chicken animal model. Anim. Microbiome.

[15] Chen Z., Zhong Y., Chen L., Liu W., Lin C., Chen Y., Wang X. (2025). HGF Aggravated Periodontitis-Associated Gut Barrier and Microbial Dysfunction: Implications for Oral–Gut Axis Regulation. Biology.

[16] Schell K.R., Fernandes K.E., Shanahan E., Wilson I., Blair S.E., Carter D.A., Cokcetin N.N. (2022). The Potential of Honey as a Prebiotic Food to Re-engineer the Gut Microbiome Toward a Healthy State. Front. Nutr..

[17] Juárez-Fernández M., Román-Sagüillo S., Porras D., García-Mediavilla M.V., Linares P., Ballesteros-Pomar M.D., Urioste-Fondo A., Álvarez-Cuenllas B., González-Gallego J., Sánchez-Campos S. (2021). Long-Term Effects of Bariatric Surgery on Gut Microbiota Composition and Faecal Metabolome Related to Obesity Remission. Nutrients.

[18] Chen Y., Chaudhari S.N., Harris D.A., Roberts C.F., Moscalu A., Mathur V., Zhao L., Tavakkoli A., Devlin A.S., Sheu E.G. (2024). A small intestinal bile acid modulates the gut microbiome to improve host metabolic phenotypes following bariatric surgery. Cell Host Microbe.

[19] Yadav J., Liang T., Qin T., Nathan N., Schwenger K.J.P., Pickel L., Xie L., Lei H., Winer D.A., Maughan H. (2023). Gut microbiome modified by bariatric surgery improves insulin sensitivity and correlates with increased brown fat activity and energy expenditure. Cell Rep. Med..

[20] Paganelli F.L., Luyer M., Hazelbag C.M., Uh H.W., Rogers M.R.C., Adriaans D., Berbers R.M., Hendrickx A.P.A., Viveen M.C., Groot J.A. (2019). Roux-Y Gastric Bypass and Sleeve Gastrectomy directly change gut microbiota composition independent of surgery type. Sci. Rep..

[21] Münzker J., Haase N., Till A., Sucher R., Haange S.B., Nemetschke L., Gnad T., Jäger E., Chen J., Riede S.J. (2022). Functional changes of the gastric bypass microbiota reactivate thermogenic adipose tissue and systemic glucose control via intestinal FXR-TGR5 crosstalk in diet-induced obesity. Microbiome.

[22] Seeley R.J., Chambers A.P., Sandoval D.A. (2015). The role of gut adaptation in the potent effects of multiple bariatric surgeries on obesity and diabetes. Cell Metab..

[23] Sabaté J.M., Coupaye M., Ledoux S., Castel B., Msika S., Coffin B., Jouet P. (2017). Consequences of Small Intestinal Bacterial Overgrowth in Obese Patients Before and After Bariatric Surgery. Obes. Surg..

[24] Amin U., Huang D., Dhir A., Shindler A.E., Franks A.E., Thomas C.J. (2024). Effects of gastric bypass bariatric surgery on gut microbiota in patients with morbid obesity. Gut Microbes.

[25] Zambrano A.K., Paz-Cruz E., Ruiz-Pozo V.A., Cadena-Ullauri S., Tamayo-Trujillo R., Guevara-Ramírez P., Zambrano-Villacres R., Simancas-Racines D. (2024). Microbiota dynamics preceding bariatric surgery as obesity treatment: A comprehensive review. Front. Nutr..

[26] Zyoud S.H., Shakhshir M., Barqawi A., Abushanab A.S., Koni A., Khilfeh S., Shahwan M., Jairoun A.A., Taha A.A., Abushamma F. (2024). Comprehensive visualization of bariatric surgery and gut microbiota research: A global analysis. Transl. Med. Commun..

[27] Cheng J., Palva A.M., de Vos W.M., Satokari R. (2013). Contribution of the intestinal microbiota to human health: From birth to 100 years of age. Curr. Top. Microbiol. Immunol..

[28] Rakab M.S., Rateb R.M., Maamoun A., Radwan N., Shubietah A., Manasrah A., Rajab I., Scichilone G., Tussing-Humphreys L., Mahmoud A.M. (2025). Impact of Probiotic/Synbiotic Supplementation on Post-Bariatric Surgery Anthropometric and Cardiometabolic Outcomes: An Updated Systematic Review and Meta-Analysis of Randomized Controlled Trials. Nutrients.

[29] de Sousa D.F., Salaroli L.B. (2024). Effects of the Use of Probiotics in Post-Bariatric Surgery Obesity: Meta-Umbrella of Systematic Reviews. Obesities.

[30] Wang Y., Zheng Y., Kuang L., Yang K., Xie J., Liu X., Shen S., Li X., Wu S., Yang Y. (2023). Effects of probiotics in patients with morbid obesity undergoing bariatric surgery: A systematic review and meta-analysis. Int. J. Obes..

[31] Belda E., Voland L., Tremaroli V., Falony G., Adriouch S., Assmann K.E., Prifti E., Aron-Wisnewsky J., Debédat J., Le Roy T. (2022). Impairment of gut microbial biotin metabolism and host biotin status in severe obesity: Effect of biotin and prebiotic supplementation on improved metabolism. Gut.

[32] Ioannou A., Berkhout M.D., Geerlings S.Y., Belzer C. (2025). Akkermansia muciniphila: Biology, microbial ecology, host interactions and therapeutic potential. Nat. Rev. Microbiol..

[33] Shen N., Caixàs A., Ahlers M., Patel K., Gao Z., Dutia R., Blaser M.J., Clemente J.C., Laferrère B. (2019). Longitudinal changes of microbiome composition and microbial metabolomics after surgical weight loss in individuals with obesity. Surg. Obes. Relat. Dis..

[34] Florent V., Dennetiere S., Gaudrat B., Andrieux S., Mulliez E., Norberciak L., Jacquez K. (2024). Prospective Monitoring of Small Intestinal Bacterial Overgrowth After Gastric Bypass: Clinical, Biological, and Gas Chromatographic Aspects. Obes. Surg..

[35] Luijten J.C.H.B.M., Vugts G., Nieuwenhuijzen G.A.P., Luyer M.D.P. (2019). The Importance of the Microbiome in Bariatric Surgery: A Systematic Review. Obes. Surg..

[36] Świerz M.J., Storman D., Staskiewicz W., Gorecka M., Jasinska K.W., Swierz A.M., Tobola P., Skuza A., Bala M.M. (2020). Efficacy of probiotics in patients with morbid obesity undergoing bariatric surgery: A systematic review and meta-analysis. Surg. Obes. Relat. Dis..

[37] Bruinsma B., Uygun K., Yarmush M., Saeidi N. (2015). Surgical models of Roux-en-Y gastric bypass surgery and sleeve gastrectomy in rats and mice. Nat. Protoc..

[38] Özkurt E., Fritscher J., Soranzo N., Hoser J., Egan L.J., Usereau N., Vandenbogaert M., Matzaraki V., Koeken V.A.C.M., Moorlag S.J.C.F.M. (2022). LotuS2: An ultrafast and highly accurate tool for amplicon sequencing analysis. Microbiome.

[39] Harris C.R., Millman K.J., Van Der Walt S.J., Gommers R., Virtanen P., Cournapeau D., Wieser E., Taylor J., Berg S., Smith N.J. (2020). Array programming with NumPy. Nature.

[40] Virtanen P., Gommers R., Oliphant T.E., Haberland M., Reddy T., Cournapeau D., Burovski E., Peterson P., Weckesser W., Bright J. (2020). SciPy 1.0: Fundamental algorithms for scientific computing in Python. Nat. Methods.

[41] Hunter J.D. (2007). Matplotlib: A 2D graphics environment. Comput. Sci. Eng..

[42] Waskom M.L. (2021). seaborn: Statistical data visualization. J. Open Source Softw..

[43] Douglas G.M., Maffei V.J., Zaneveld J.R., Yurgel S.N., Brown J.R., Taylor C.M., Huttenhower C., Langille M.G.I. (2020). PICRUSt2 for prediction of metagenome functions. Nat. Biotechnol..

