# Supplementary material for: Site-Specific Gut Microbiome Changes After Roux-en-Y Gastric Bypass in Rats: Effects of a Multicomponent Bovine Colostrum-Based Complex
Source: Int J Mol Sci. 2025 Jul 25;26(15):7186. doi: 10.3390/ijms26157186 (PMC12347457; doi:10.3390/ijms26157186)
Supplement: Supplementary file 1 [file ijms-26-07186-s001.zip › ijms-3759010-supplementary.pdf]

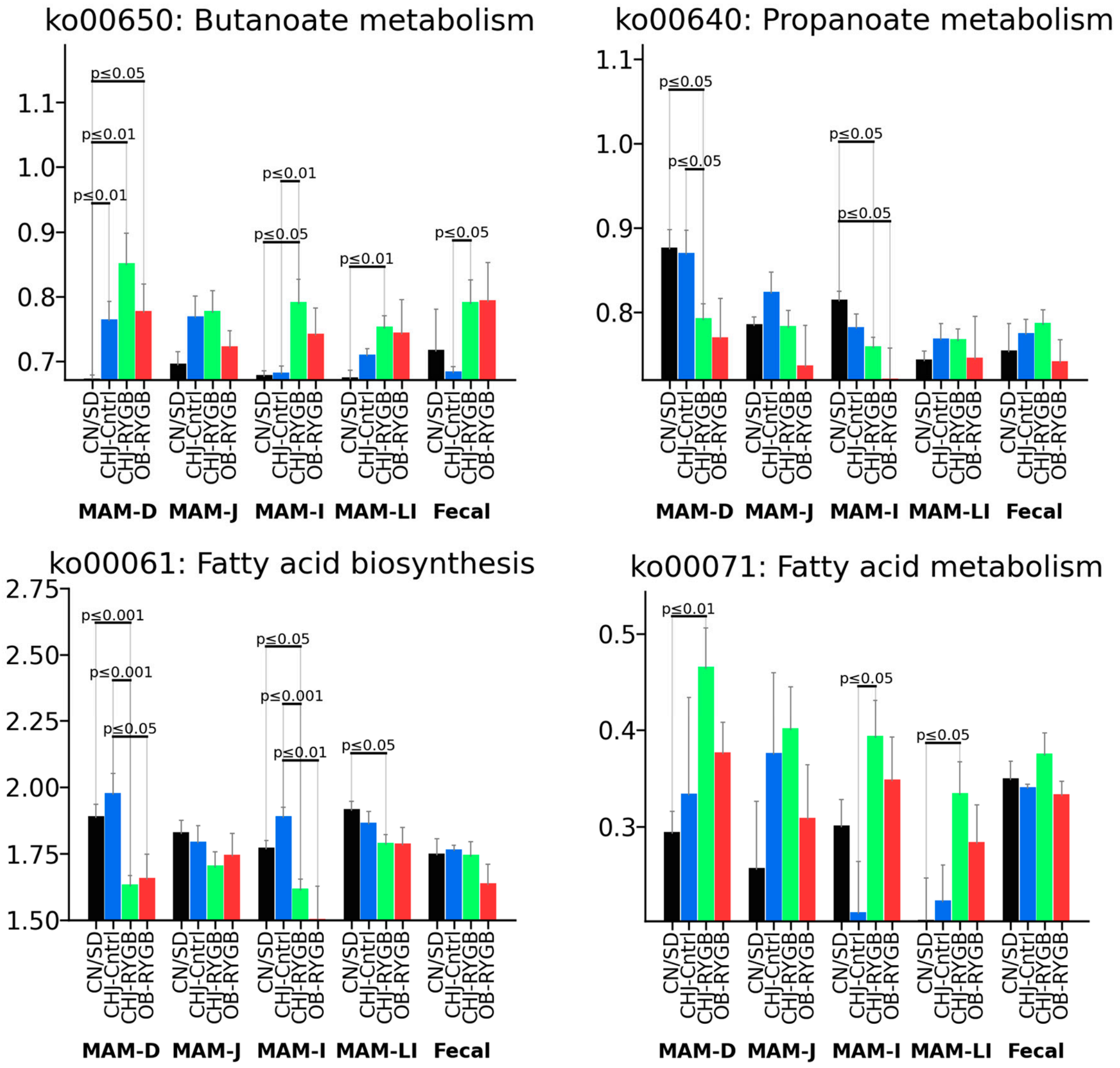

Supplement Figure S1. Predicted short-chain fatty acid production capacity remains stable despite taxonomic shifts. PICRUSt2 functional prediction showing that while SCFA-producing bacterial abundance decreased significantly, the metabolic potential for SCFA production remained largely unchanged, suggesting compensatory mechanisms or methodological limitations of predictive analysis.
